# Impaired ex vivo T-cell expansion before leukapheresis is associated with out-of-specification tisagenlecleucel manufacturing in pediatric CD19^+^ALL

**DOI:** 10.3389/fimmu.2026.1973579

**Published:** 2026-10-27

**Authors:** Melanie Bremm, Greta Heinze, Lara Schneeberger, Emilia Salzmann-Manrique, Julia Banisharif Dehkordi, Andreas Peinelt, Krzysztof Kalwak, Monika Mielcarek-Siedziuk, Roland Meisel, Rita Beier, Claudia Rossig, Andrea Jarisch, Juliane Schlueter, Eva Rettinger, Jan-Henning Klusmann, Claudia Cappel, Peter Bader, Sabine Huenecke

**Affiliations:** 1 Department of Pediatrics, Goethe-University Frankfurt, Frankfurt, Germany; 2 Department of Hematology and Oncology, University Medical Center of the Johannes Gutenberg University, Mainz, Germany; 3 Department of Pediatric Hematology/Oncology and BMT, Wroclaw Medical University, Wroclaw, Poland; 4 Department of Pediatric Oncology, Hematology and Clinical Immunology, Division of Pediatric Stem Cell Therapy, Medical Faculty, Heinrich Heine University, Duesseldorf, Germany; 5 Pediatric Hematology and Oncology, Hannover Medical School, Hannover, Germany; 6 Department of Pediatric Hematology and Oncology, University Children’s Hospital Münster, Münster, Germany

**Keywords:** apheresis, CAR-T cells, children, T-cell proliferation, tisagenlecleucel

## Abstract

**Introduction:**

Chimeric antigen receptor (CAR)-T cell therapy requires individual production for each patient, starting with a heterogeneous apheresis product of variable cellular composition and function. This inter-patient variability presents a major challenge for standardizing manufacturing processes and may lead to delays or even production failure. The primary aim of this study was to identify pre-leukapheresis characteristics associated with subsequent manufacturing deviations.

**Methods:**

Multiple T cell-related parameters including T cell receptor (TCR) Vβ repertoire, differentiation status, proliferation capacity (e.g. fraction diluted – DIL), and exhaustion were examined in n = 27 patients before leukapheresis for Kymriah^®^ immunotherapy. These parameters were analyzed for associations with manufacturing specification compliance and out-of-specification (OOS) events.

**Results:**

Impaired T-cell proliferation was the parameter most strongly associated with manufacturing specification non-conformance. Six of 21 evaluable patients showed poor proliferation (DIL <80%), including all four patients with OOS manufacturing outcomes. In contrast, all remaining patients had DIL values ≥84.9%. Exploratory associations between selected TCR Vβ subsets and manufacturing specifications were also identified.

**Conclusion:**

DIL was the parameter most strongly associated with manufacturing specification compliance and may represent a simple functional measure of pre-apheresis T-cell fitness.

## 1 Introduction

Tisagenlecleucel (Kymriah^®^) is a CD19-directed CAR-T cell therapy that induces durable responses in pediatric patients with relapsed or refractory ALL (1–5). The quality of the starting material is a critical determinant of successful CAR-T cell manufacturing. Previous studies have shown that T-cell differentiation status, proliferative capacity, and leukapheresis characteristics can influence manufacturing outcomes and subsequent clinical responses (1–9). However, most available studies focused on material collected at the time of leukapheresis or on the leukapheresis product itself (6–9). Data obtained before leukapheresis, particularly in pediatric patients, remain limited.

To manufacture tisagenlecleucel, autologous T cells must be collected via leukapheresis, cryopreserved, and transported to a centralized production facility. CAR-T cell production is a complex, resource-intensive process involving viral transduction of T cells with a second-generation CAR containing the CD3ζ signaling domain and 4-1BB as co-stimulatory module. The modified T cells are then expanded ex vivo to reach the therapeutic cell dose (1, 4, 5). Manufacturing is performed in accordance with Good Manufacturing Practice (GMP) conditions. CAR-T cell products are classified by the European Medicines Agency (EMA) as Advanced Therapy Medicinal Products (ATMPs) (2, 10). Quality control prior to administration is essential, as even minor deviations in production can significantly impact therapeutic efficacy and safety (11). The autologous nature of CAR-T cell therapy entails individualized production for each patient, starting with heterogeneous apheresis products of variable cellular composition and function (12). This inter-patient variability, further exacerbated by prior treatments such as hematopoietic stem cell transplantation (HSCT), presents a major challenge for standardizing manufacturing processes (7). Poor cell numbers and quality of the starting material may lead to treatment delays or even production failure.

CAR-T cell manufacturing outcomes generally fall into one of three categories (1): compliant manufacturing, where the final product meets all specifications (2); manufacturing failure, where no product can be generated; and (3) non-compliant manufacturing, where a product is generated but does not fully meet the pre-defined specification criteria, leaving the decision of administration to the treating physician.

As CAR-T cell therapy expands to new indications and earlier treatment lines, the number of eligible patients continues to rise (13, 14).

We hypothesized that functional and phenotypic characteristics measured before leukapheresis may be associated with subsequent manufacturing outcomes. For this purpose, assays were established to assess T cell differentiation, T cell exhaustion, T cell Vβ repertoire, and proliferative capacity in patients with and without HSCT collected prior to leukapheresis. These parameters were subsequently analyzed in relation to the product specifications.

## 2 Materials and methods

### 2.1 Patients

This study included patients under the age of 25 who were treated with Kymriah^®^ for relapsed or refractory CD19+ B-cell leukemia. A total of 27 patients from five centers participated in the study. Patients were treated at the following University hospitals: Frankfurt (n = 18), Dusseldorf (n = 1), Munster (n = 2), and Hanover (n = 3) in Germany, as well as Wroclaw (n = 3) in Poland.

Of the 27 enrolled patients, two died during the study period, one apheresis was unsuccessful, and one patient relocated and was lost to follow-up. Therefore, data from 23 patients were available for analysis, of whom 17 had previously received HSCT. The CAR-T cells were administered a median of 422 days after HSCT (range: 83–814 days) with only three patients needing early CAR-T cell intervention within the first six months post-HSCT. Patient characteristics are summarized in **Table 1**. Active GvHD was an exclusion criterion for leukapheresis and CAR T-cell manufacturing. Accordingly, only one patient in the cohort had a history of grade 1 GvHD, while no patients had active moderate or severe GvHD.

**Table 1:** Patient characteristics.

No	Center	sex	SCT	Age at apheresis	Days post SCT	ALC (nl)	CD3 (µl)	CAR production	QC specifications	Differentiation	TCRVβ	Exhaustion	Proliferation
1	F	f	yes	3.0	87	9.27	3273	yes	OOS	yes	yes	no	yes
2	F	m	no	14.5	N.A.	6.13	1023	no	N.A.	yes	yes	no	no
3	F	f	no	10.3	N.A.	3.28	641	yes	fulfilled	yes	yes	no	yes
4	F	m	no	15.2	N.A.	8.87	751	yes	OOS	yes	yes	yes	yes
5	F	m	yes	16.8	814	12.36	4004	yes	fulfilled	yes	yes	yes	yes
6	D	f	yes	2.5	85	4.2	603	yes	fulfilled	yes	yes	yes	yes
7	M	f	yes	9.3	793	5.67	2227	yes	fulfilled	yes	yes	yes	yes
8	H	m	yes	1.1	69	3.6	65	no	N.A.	no	no	no	no
9	F	m	yes	14.9	224	3.81	408	no	N.A.	yes	yes	yes	no
10	F	m	yes	8.9	561	5.91	2060	yes	OOS	yes	no	yes	yes
11	F	m	yes	14.9	387	3.28	1127	yes	fulfilled	yes	yes	yes	yes
12	F	m	no	13.2	N.A.	1.58	758	yes	fulfilled	yes	yes	yes	no
13	F	m	yes	10.2	500	2.51	1663	yes	fulfilled	yes	yes	yes	no
14	F	m	yes	8.8	409	5.66	2115	yes	fulfilled	yes	yes	yes	yes
15	M	m	no	17.3	N.A.	3.6	596	yes	fulfilled	yes	yes	yes	yes
16	F	f	no	7.0	N.A.	3.06	325	yes	fulfilled	yes	yes	yes	yes
17	F	f	yes	8.4	422	2.18	505	yes	fulfilled	yes	yes	yes	yes
18	H	f	yes	17.1	759	9.9	403	yes	fulfilled	yes	yes	yes	yes
19	F	m	yes	15.7	378	5.89	2444	yes	fulfilled	yes	yes	yes	yes
20	F	f	no	12.0	N.A.	1.7	715	yes	OOS	yes	yes	yes	yes
21	F	m	no	6.3	N.A.	3.68	1142	yes	fulfilled	yes	yes	yes	no
22	F	m	no	10.4	N.A.	5.28	2926	no	N.A.	yes	yes	yes	yes
23	H	f	yes	1.9	400	7.2	2392	yes	fulfilled	yes	yes	yes	yes
24	F	f	yes	6.3	502	2.11	1229	yes	fulfilled	yes	yes	yes	yes
25	W	m	no	17.4	N.A.	1.24	362	yes	fulfilled	yes	yes	yes	yes
26	W	f	yes	4.7	196	5.47	1058	yes	fulfilled	yes	yes	yes	yes
27	W	f	yes	8.7	1483	20.99	3230	yes	fulfilled	yes	yes	yes	yes

Written informed consent was obtained from all patients or their legal guardians, and the study was approved by the relevant ethics committee (approval number 20-807).

### 2.2 Study design

Blood samples from study participants were collected within seven days before leukapheresis. All blood punctures were performed as part of routine examinations to monitor the patient’s condition. No additional punctures, hospitalizations or follow-up examinations were necessary as a result of participation in the study.

### 2.3 Product specifications

Kymriah product specifications are evaluated across the following categories A) Purity: Cell viability and Percent viable cells, B) Quantity: Number of viable cells, C) Potency: IFN-γ release and CAR expression. A product which fails to meet one or more of the defined specifications or sterility testing, is classified as Out-of-Specification (OOS).

### 2.4 Analyses before leukapheresis

#### 2.4.1 T cell differentiation and exhaustion

Peripheral blood (PB) samples were collected in EDTA tubes, kept at room temperature (RT), and analyzed within 24 hours. Flow cytometric analysis of immune cell subsets was performed as dual-platform approach analysis using a five-color FC500 or Navios™ flow cytometer (Beckman Coulter, Krefeld, Germany).

The following fluorochrome-conjugated monoclonal antibodies were used: CD3 (UCHT1), CD4 (SFCI12T4D11), CD8 (SFCI21Thy2D3), CD45RA (ALB11), CD45RO (UCHL1), CD62L (DREG56) (Beckman Coulter, Immunotech, Marseille, France). Quantification was performed for total CD3^+^ T cells, as well CD3^+^CD4^+^ (T4) and CD3^+^CD8^+^ (T8) subsets. The differentiation was assessed within the T4 and T8 compartments, identifying the following subsets: naive (CD45RA^+^CD62L^+^, naive T cells), central memory (CD45RO^+^CD62L^+^, CM T cells), effector memory (CD45RO^+^CD62L^-^, EM T cells), and effector memory RA-positive (CD45RA^+^CD62L^-^, EMRA T cells).

For T cell exhaustion analysis, the following reagents were used: PD-1 (AD2), TIM-3 (7D3), CD19 (J3-119), 7AAD (viability), CD56 (N901), CD3 (UCHT1), CD8 (B9.11), CD4 (13B8.2), LAG-3 (T47-530), and CD45 (J33).

#### 2.4.2 TCR Vβ repertoire

The Vβ repertoire was analyzed using the Beta Mark TCR Vβ Repertoire Kit (Beckman Coulter, Marseille, France), which enables the detection of 24 different Vβ families using eight reagent cocktails (15, 16). Each reagent cocktail covers three distinct Vβ regions, represented by either FITC, PE, or a combination of both fluorochromes. For each tube, 20µL of the respective reagent cocktail was added to 50µL of EDTA-PB. The following monoclonal antibodies were included for immunophenotyping: CD3 (UCHT1), CD4 (13B8.2), CD8 (SFCI21Thy2D3) and CD45 (J33) with ECD, PC5, PC7 and KO, respectively.

#### 2.4.3 T cell proliferation

Peripheral blood (PB) samples (≥7 mL) collected in lithium heparin tubes were subjected to Ficoll density gradient centrifugation to isolate peripheral blood mononuclear cells (PBMCs). As a fluorescence-minus-one (FMO) control, 0.5 × 10^6^ unstained but stimulated with anti-CD3 and anti-CD28 PBMCs were harvested, washed (7 min at 350 × g), resuspended in 0.5 mL RPMI-1640 medium (Gibco, Thermo Fisher Scientific, Waltham, MA, USA), supplemented with 10% fetal calf serum (FCS; Merck, Darmstadt, Germany), and transferred to a 48-well plate (Corning, Glendale, USA).

For the proliferation assay, 5 × 10^6^ PBMCs were stained with 1 µL Violet Proliferation Dye 450 (VPD450; BD Biosciences, San Jose, USA). Three aliquots of 0.5 × 10^6^ cells were prepared from the stained PBMCs: 1.Unstimulated/stained (baseline, day 0), 2.Stimulated/stained (day 4). 3.Unstimulated/stained (day 4). The FMO and stimulated samples were additionally incubated with 0.2 µL of both anti-CD3 and anti-CD28 antibodies (Ultra-LEAF™, BioLegend, San Diego, USA) for T cell activation.

Flow cytometric analysis was performed on day 0 (unstimulated control) and day 4 (stimulated and unstimulated samples) using a BD Lyric flow cytometer (BD Biosciences, Heidelberg, Germany) with the following antibodies: CD45 (J33), 7-AAD, CD4 (SFCI12T4D11), CD8 (SFCI21Thy2D3), and CD5 (L17F12), respectively.

Data were analyzed using FlowJo™ software (BD Biosciences, San Jose, USA). Several established proliferation parameters were evaluated (1): Dil (fraction diluted): the proportion of cells that have undergone at least one division (2). PD/PF (percent divided/precursor frequency): the probability that a cell from the original population has divided at least once (3). EI (expansion index): indicates the fold increase of the total cell population (e.g., an EI of 3.0 reflects a threefold expansion) (4). PI (proliferation index): the average number of divisions among responding cells only (17) (5). Peaks: the number of discernible proliferation peaks, corresponding to cell divisions.

The DIL assay was validated according to ICH Q2(R2). Repeatability (intra-assay CV) was 2.1% and intermediate precision (inter-assay CV) was 7.9%, determined from a positive control measured in triplicate in six independent runs. To establish a reference range, peripheral blood samples from 13 healthy donors were analyzed using the same protocol (mean 92.8% ± 4.4% SD, range 84.3–97.6%). Poor proliferation was defined as a DIL below the mean minus three standard deviations of the healthy donors (79.6%), i.e. <80%.

### 2.5 Statistical analysis

Statistical analyses were performed using GraphPadPrism 10 (GraphPad Software, San Diego, USA) and R version 4.3.1. P-values <0.05 were regarded significant and indicated by *(<0.05), **(<0.01), ***(<0.001) and ****(<0.0001).

Univariate analyses were performed using Spearman’s rank correlation. Multivariate analyses were conducted using linear regression with backward stepwise variable selection, complemented by random forest algorithms.

In the random forest analysis, only variables that did not correlate with each other (Spearman correlation <0.4) were included. The IncMSE parameter stands for ‘Increase in Mean Squared Error’ and is used to evaluate the importance of an individual feature in the random forest model.

## 3 Results

### 3.1 Cohort characteristics and manufacturing outcomes

T cell differentiation, TCR repertoire, T cell exhaustion, and T cell proliferation capacity were analyzed in patient samples collected within seven days prior to leukapheresis. Data for analysis was available from 23/27 patients. In four cases (patients #2, #8, #9, and #22) no CAR-T cell product could be generated due to production failures (n=2), insufficient T cell collection and non-payment of costs. Moreover, one product failed the viability criterion, one did not meet the CAR expression threshold, and one product failed both viability and INF-γ release product specifications. An additional product was OOS due to an invalid mycoplasma polymerase chain reaction (PCR) result. Despite these manufacturing deviations, all products were administered after individual clinical assessment. In all cases, the OOS status was communicated to the patients and their parents/legal guardians, and the products were released by the manufacturer on this condition. The decision to infuse was based on the minor nature of the deviations and on the delay of approximately 4 to 6 weeks that re-manufacturing would have caused. Of note, CAR T cells expand *in vivo* upon target antigen recognition, so that expansion can be expected even with low initial CAR expression; this release specification therefore does not necessarily reflect *in vivo* efficacy. Infusion did not preclude a second leukapheresis and manufacturing attempt in case of insufficient clinical response.

Therefore, the focus of the present study was not to distinguish infused from non-infused products, but to explore factors associated with manufacturing specification compliance. Manufacturing specification non-conformance was therefore used as a surrogate manufacturing endpoint rather than a direct measure of clinical efficacy.

### 3.2 T cell composition before leukapheresis in patients with and without prior HSCT

No significant differences in total CD3^+^ T cell counts were observed between patients with and without prior HSCT, although CD4^+^ T cell counts were significantly higher in the HSCT group (p = 0.046; **Figure 1A**). Among T cell subsets, HSCT patients showed higher absolute CD4^+^ EMRA T cell counts (p = 0.011), whereas CD4^+^ central memory T cells were more prevalent in non-HSCT patients (p = 0.012). No differences were observed in CD8^+^ T cell subsets (**Figure 1B**). Median absolute CD3^+^ T-cell counts were more than twice as high in patients with prior HSCT (n = 17) as in patients without prior HSCT (n = 10) (1663 cells/µL, range: 65–4004 vs. 733 cells/µL, range: 325–2926; p = 0.10). This trend was attributable to higher counts of both CD4^+^ T cells (553 cells/µL, range: 28–2083 vs. 340.5 cells/µL, range: 184–611; p = 0.10) and CD8^+^ T cells (764 cells/µL, range: 4–2694 vs. 325.5 cells/µL, range: 135–2357; p = 0.19). None of these differences reached statistical significance (Mann–Whitney test; **Figure 1A**; **Supplementary Figure S2**).

**Figure 1:**
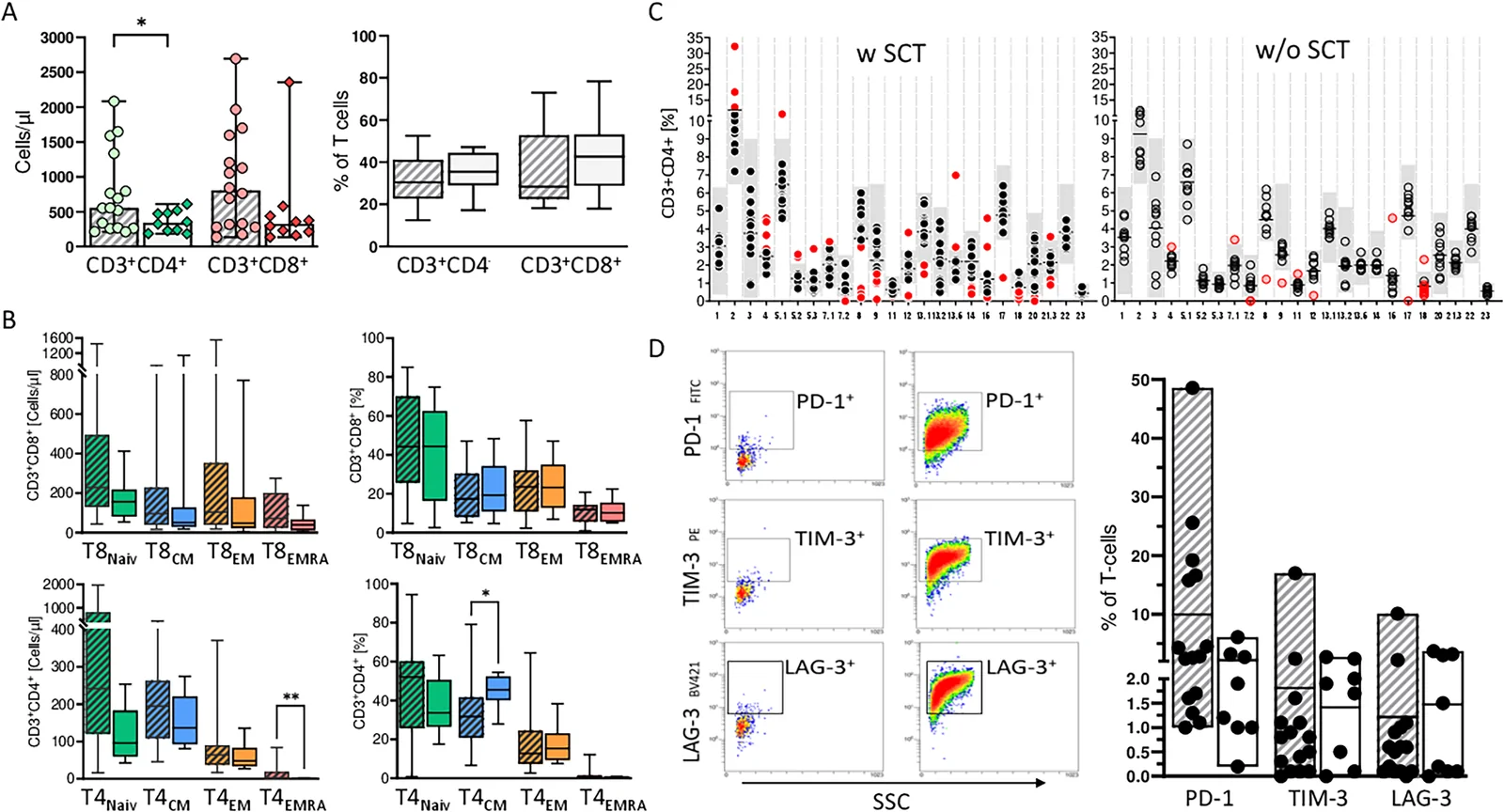
T cell composition before leukapheresis in patients with and without prior HSCT. T cell differentiation, Vβ repertoire and T cell exhaustion within seven days before leukapheresis for CAR-T cell generation are shown for patients with (striped bars) and without prior HSCT (clear bars). Comparison of **(A)** T helper (T4) cells and cytotoxic (T8) T cells, and **(B)** T cell differentiation subsets before leukapheresis (n = 26 patients). Subsets are displayed as absolute cell counts (left) and as percentage of T cells (right). Whisker plots indicate median, interquartile range, and minimum/maximum. **(C)** Measurements of Vβ expression on T4 and T8 cells (n=25 patients). The graph shows the percentage of each Vβ family in individual study patients. Gray area indicates the reference range of the Vβ subset. Results out of the reference range are colored in red. **(D)** Representative flow cytometry measurements of PD-1, TIM-3, and LAG-3 (left). Exhaustion parameters PD-1, TIM-3, and LAG-3 before CAR-T cell apheresis (right) (n = 23 patients). Box plots represent mean, minimum and maximum. * represents statistical significance (s. section 2.5).

TCR Vβ analysis revealed significantly more receptors outside the reference range among CD4^+^ T cells in HSCT patients compared with non-HSCT patients (17% vs. 8%; p = 0.0049; **Figure 1C**), indicating altered repertoire distribution after HSCT. Vβ18 was frequently underrepresented in both groups. PD-1 expression tended to be higher following HSCT, while TIM-3 and LAG-3 expression showed greater variability (**Figure 1D**).

Proliferation assay data were available for 22 patients (HSCT: n = 14; non-HSCT: n = 8). An exemplary proliferation analysis is shown in **Figure 2A**. The HSCT and non-HSCT groups displayed no significant differences in their median value for fraction diluted (DIL), precursor frequency (PF), expansion index (EI), and proliferation index (PI), suggesting an overall comparable proliferation capacity.

**Figure 2:**
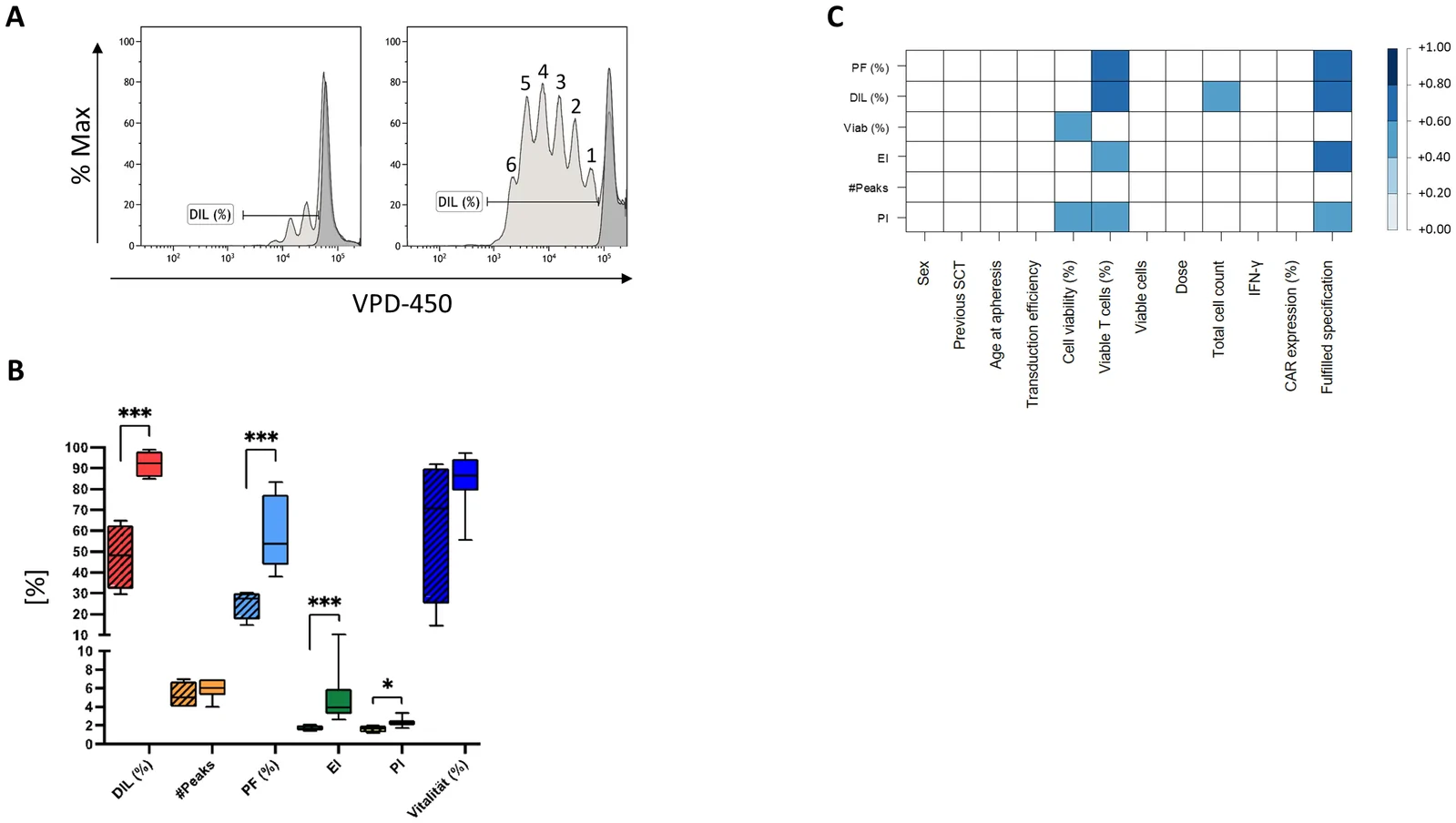
Relationship between proliferation parameters and product specifications. **(A)** Representative proliferation plots showing limited (left) and strong (right) T-cell proliferation after 4 days of stimulation. **(B)** Comparison of proliferation parameters between the groups with OOS results (n = 4, striped bars) and without OOS results (n = 16, clear bars, all product specifications fulfilled). Whisker plots indicate median, interquartile range, and minimum/maximum. The following proliferation parameters were evaluated: (a) DIL (fraction diluted): the proportion of cells that have undergone at least one division. (b) Peaks: the number of discernible proliferation peaks, corresponding to cell divisions. (c) PF (precursor frequency): the probability that a cell from the original population has divided at least once. (d) EI (expansion index): indicates the fold increase of the total cell population (e.g., an EI of 3.0 reflects a threefold expansion). (e) PI (proliferation index): the average number of divisions among responding cells only. (f) Viability on the basis of 7-AAD uptake. **(C)** Heatmap representation of the relationship between proliferation parameters and specification parameters.

### 3.3 Correlation of T cell parameters with product specifications

T cell differentiation subsets — including naïve, central memory (CM), effector memory (EM), and effector memory RA-positive (EMRA) cells —were analyzed separately for both CD4^+^ and CD8^+^ T cell compartments. Correlations between these subsets and product specifications are summarized in **Figure 3A**. Moderate negative correlations were observed between total CD3^+^ T cells, cytotoxic T cells, and specifically CD8^+^ EMRA T cells and the total cell count in the CAR-T cell product. Additionally, CD8^+^ EMRA T cells showed a moderate negative correlation with cell viability. In contrast, EMRA CD4^+^ T cells were moderately positively correlated with prior HSCT, indicating a more differentiated phenotype in this subgroup. However, no significant correlation was found between any T cell differentiation subset and overall specification fulfillment.

**Figure 3:**
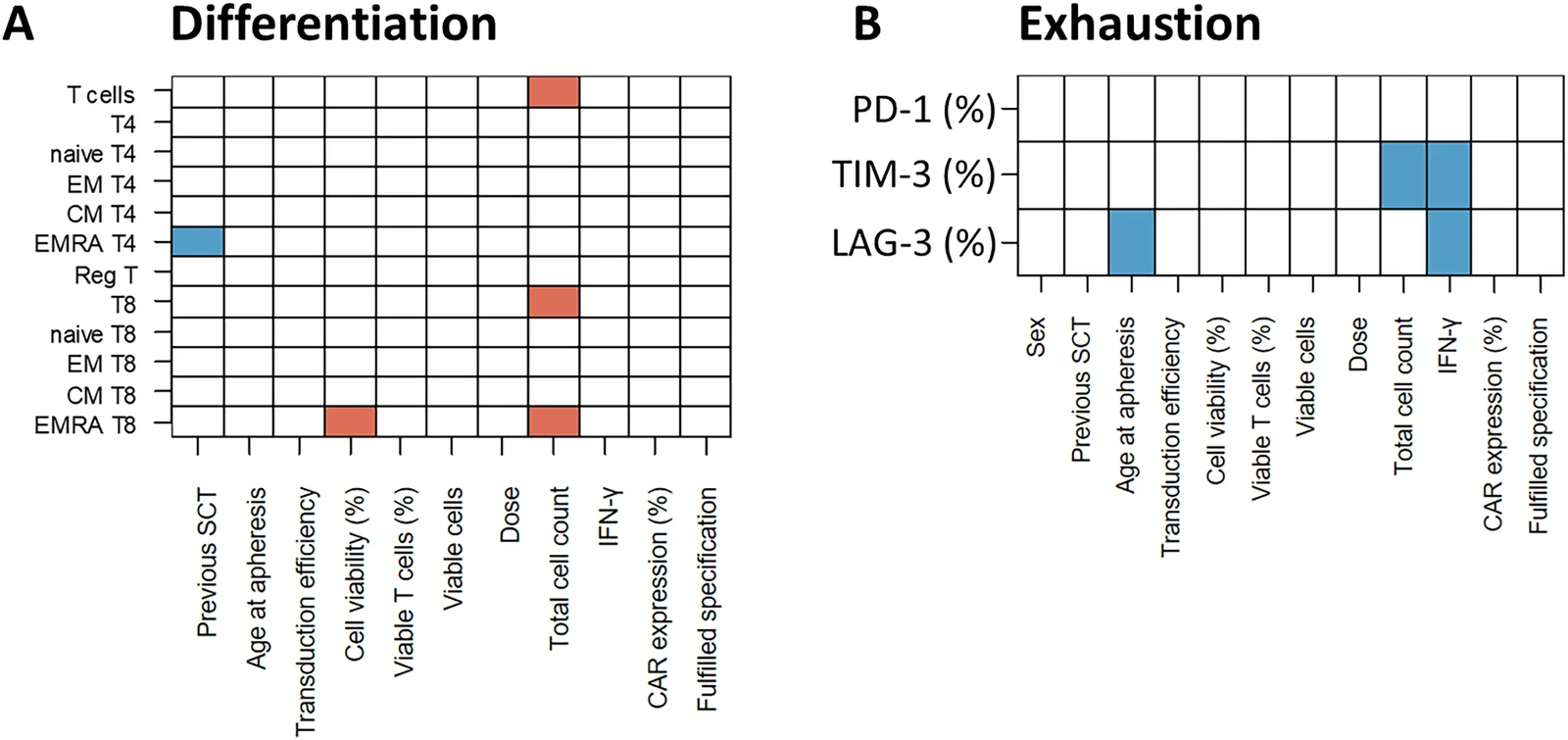
**(A)** Correlation matrix for T cell differentiation and product specifications (n = 23 patients). **(B)** Correlation of the exhaustion markers PD-1, TIM-3 and LAG-3 with CAR-T cell product specifications (n = 21 patients).

Furthermore, T cell exhaustion status at the time of leukapheresis was evaluated in relation to product specifications (**Figure 3B**). Moderate negative correlations were found between TIM-3 expression and both total cell count and IFN-γ release, while LAG-3 expression was negatively correlated with IFN-γ release and patient age. PD-1 expression was consistently detectable among patients but showed no significant associations.

Proliferation analysis revealed significantly higher values of the parameters DIL, PF, EI, and PI in T cells from patients without OOS results than from patients with OOS results (**Figure 2B**). Six of 21 evaluable patients (29%), including all four patients with OOS products, showed poor proliferation (DIL 29.6-75.8%), whereas all remaining patients had DIL values ≥84.9%. T cell proliferation parameters were correlated with product specifications as shown in **Figure 2C**. A clear positive correlation was observed between PF and DIL with the percentage of viable cells, suggesting that robust proliferation is associated with better product quality. Most notably, PF, DIL, and EI were strongly positively correlated with overall specification conformance, supporting an association between pre-apheresis T-cell proliferative capacity and manufacturing outcomes.

Vβ subset frequencies in CD4^+^ and CD8^+^ T cells were correlated with individual CAR-T cell product specifications and overall specification conformance. The number of Vβ subsets outside the established reference range was quantified as T4 out (CD4^+^) and T8 out (CD8^+^; **Supplementary Figure 1**). Strong negative correlations were observed between T8 out and cell viability, and between CD4^+^ Vβ21.3 expression and final product dose (r = –0.6 to –0.8). Moderate negative correlations with overall specification conformance were identified for CD4^+^ Vβ1, Vβ4, Vβ5.2, and Vβ20, as well as CD8^+^ Vβ2 (CD8^+^; **Supplementary Figure 1**).

### 3.4 Multivariate analysis of T cell parameters with [roduction results

**Figure 4** summarizes the most relevant findings of the multivariate analysis in relation to the specification categories: purity, quantity, potency, and overall specification conformance. For purity, the parameters cell viability and percentage of viable cells were analyzed (**Figure 4A**). The results of the proliferation assay (particularly PI and DIL), as well as the frequency of CD4^+^ T cells, showed the most pronounced influence on these parameters. The category quantity was assessed by measuring the absolute number of viable cells in the CAR-T cell product (**Figure 4B**). The random forest analysis identified TCR Vβ14 expression on CD8+ T cells as one of the variables most strongly associated with the number of viable cells. A decision tree revealed that if Vβ14 expression on CD8^+^ T cells was ≤ 5.2%, the total viable cell count typically ranged between 0 and 1 × 10^9^ cells. Expression levels above this threshold were associated with significantly higher cell counts. Regarding potency, the parameters IFN-γ release and CAR expression were analyzed. The highest variable importance for IFN-γ release was shown for Vβ7.2 expression on CD8^+^ T cells, followed by the overall proportion of CD4^+^ T cells (**Figures 4C**, left). For CAR expression, the frequency of cytotoxic EMRA T cells and the PF from the proliferation assay emerged as the most informative variables. A decision tree based on EMRA CD8^+^ T cell counts demonstrated that patients with > 59 CD8^+^ EMRA T cells/μL before apheresis (**Figures 4C, right**) showed significantly higher CAR expression in their products (p = 0.005). Lastly, most strongly associated variables for overall product quality — i.e., conformance with all specification criteria without OOS events—were assessed. Here, PF showed the strongest association with overall specification conformance (**Figure 4D**). After exclusion of the product classified as OOS solely due to an invalid mycoplasma PCR result, the association between impaired proliferation and OOS manufacturing persisted in the permutation-based analysis, although the corresponding %IncMSE decreased to 3.63.

**Figure 4:**
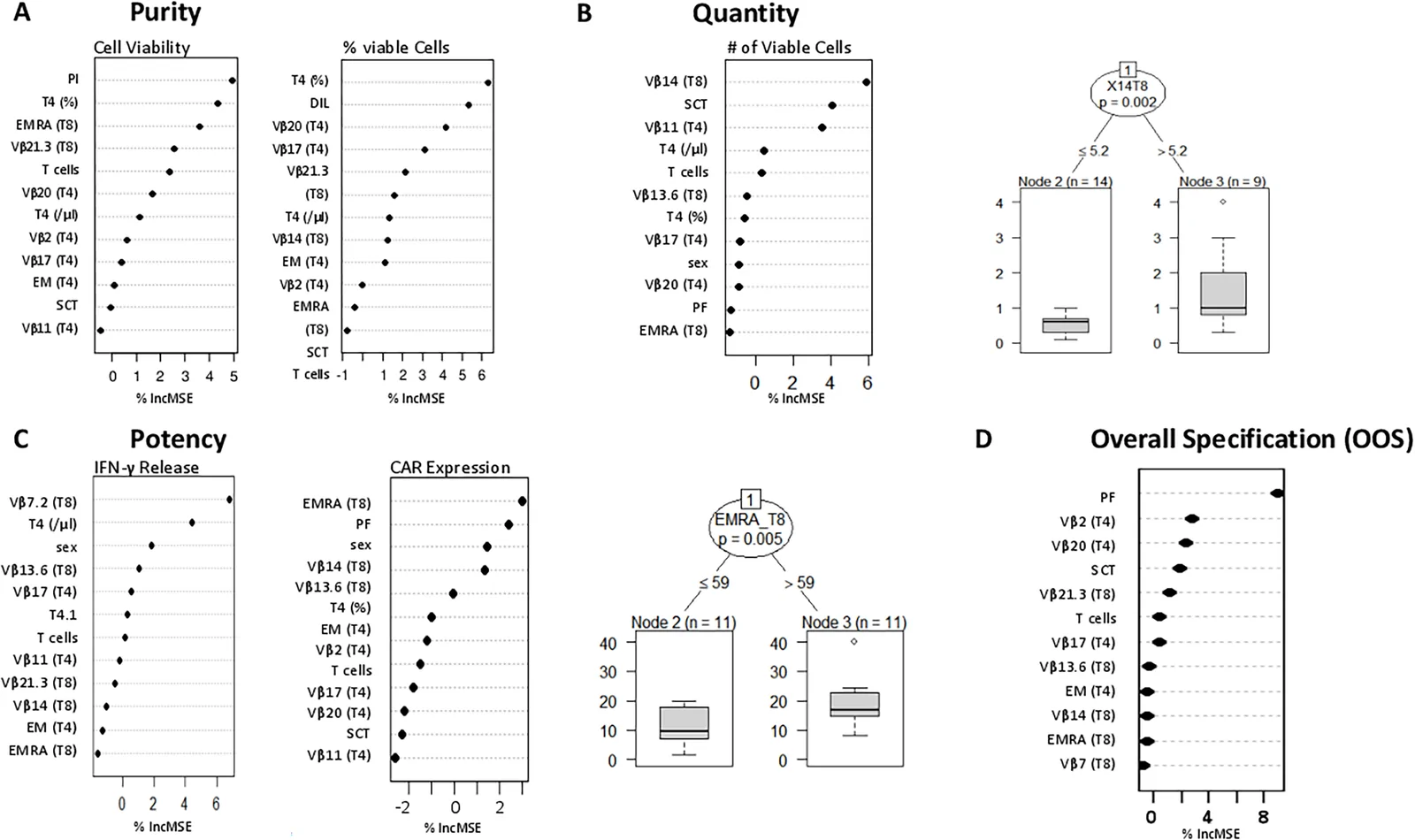
Multivariate analyses of T cell parameters with CAR-T cell specifications. Shown are random forest analysis results and selected decision trees for the product specification categories purity, quantity, potency, as well as for the fulfillment of all specifications (no OOS) for n = 17 patients. The plots display the IncMSE (Increase in Mean Squared Error), which serves as a measure of importance of a variable, as it represents the percentage increase in prediction error observed when each variable is removed from the random forest analysis. The models were set up excluding the variables that intercorrelated significantly. **(A)** Purity: Random forest analysis plots for the specifications ‘cell viability’ and ‘percent viable cells’. **(B)** Quantity: Random forest plot for the specification ‘number of viable cells’ (left) and decision tree visualization for the parameter TCR Vβ14 as a percentage of T8 cells (right). TCR Vβ14 showed the strongest association with this specification in the random forest analysis. If TCR Vβ14 is detectable on less than or equal to 5.2% of cytotoxic T cells, the absolute number of viable T cells in the CAR product is in the range of 0–1 x 10^9^. If Vβ14 expression is detectable on more than 5.2% of the cytotoxic T cells, the total number of viable cells was significantly higher (p = 0.002). **(C)** Potency: Random forest results for the specification IFN-γ release and CAR expression (left). Additionally, a decision tree is shown, demonstrating significantly higher CAR expression in products from patients with >59 CD8^+^ EMRA cells/µL (right). **(D)** Overall Specification: The precursor frequency (PF; proliferation analysis) showed the strongest association with overall specification conformance.

### 3.5 Variables associated with CAR-T cell specification conformance

The product specifications along with the most informative variables identified are summarized in **Figure 5**. TCR Vβ subsets and proliferation parameters showed associations in correlation and random forest models. Among the 24 Vβ subsets analyzed, TCR Vβ20 (CD4^+^ T cells) was among the variables most consistently associated with negative association for both of the purity parameters as well as overall specification conformance. In the random forest analysis, TCR Vβ14 (CD8^+^ T cells) and TCR Vβ7.2 (CD8^+^ T cells) showed notable associations for the number of viable cells and IFN-γ release, respectively. Within T cell differentiation subsets, the proportion of CD4^+^ helper T cells were associated with both cell viability and the percentage of viable T cells. Moreover, cytotoxic EMRA T cells (CD8^+^) exhibited strong association with the capacity for CAR expression.

**Figure 5:**
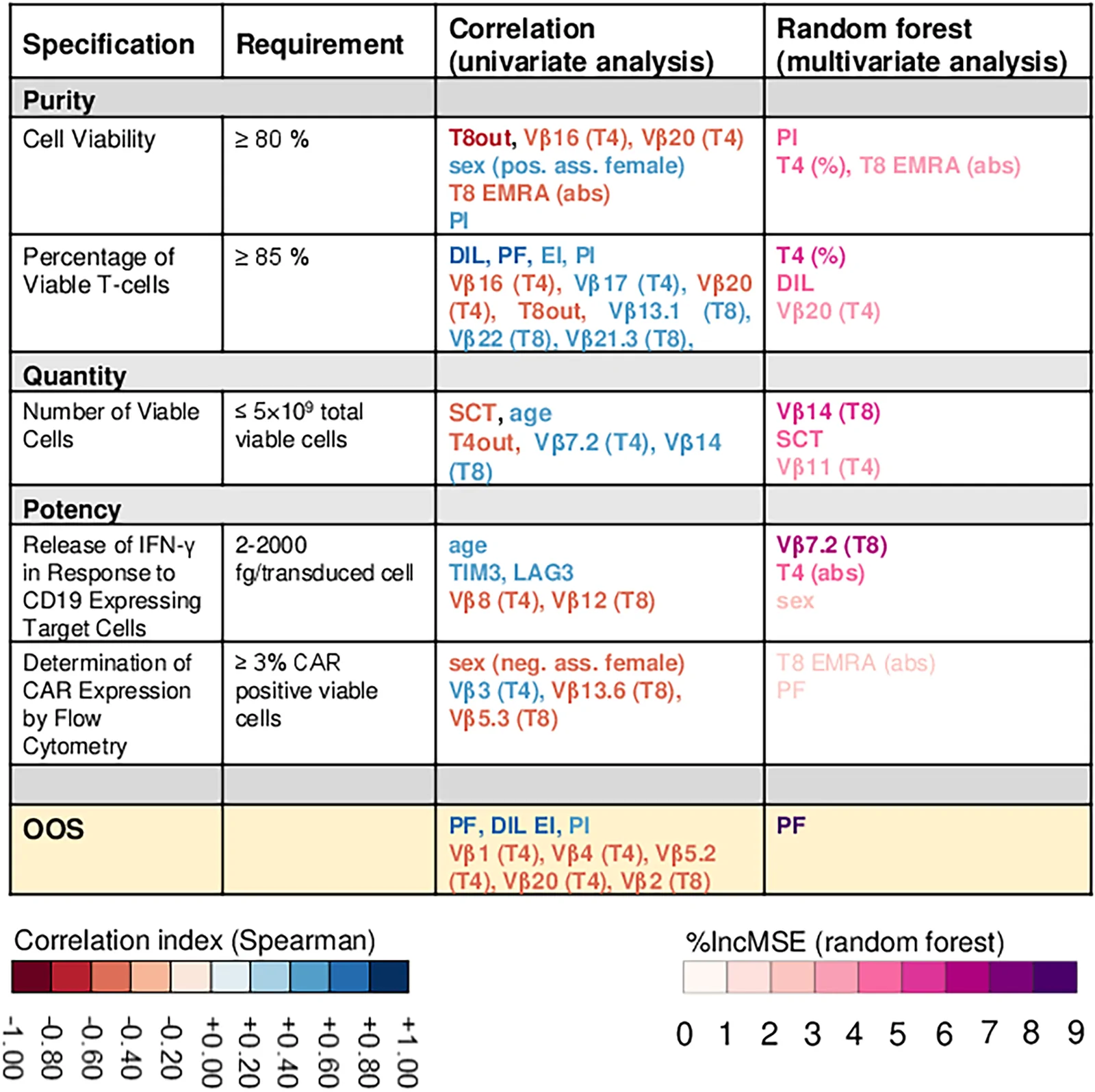
Variables associated with CAR-T cell manufacturing specifications. The specifications considered (rows) and the parameters showing the strongest associations in the univariate analysis (Spearman correlation) and the multivariate random forest analysis (columns) are shown. For univariate analyses, negative correlations are indicated in red and positive correlations in blue, color intensity corresponds to the magnitude of the correlation. For multivariate analyses, darker shades represent higher percentages of IncMSE. IncMSE stands for ‘Increase in Mean Squared Error’. This value describes the relative importance of a variable within the random forest model.

Among all parameters, proliferation markers showed the strongest association with Out-of-Specification (OOS) events. Poor T cell proliferative capacity was strongly associated with failure to meet all specifications. We observed a clear correlation between multiple proliferation assay metrics (DIL, PF, EI, PI) and CAR-T cell product quality, particularly in relation to the purity criteria. Among them DIL may serve as a single proliferation parameter, streamlining the analysis process and offering a practical, software-independent metric for identifying variables associated with CAR-T cell manufacturing success.

## 4 Discussion

Identifying variables associated with unsuccessful CAR-T cell manufacturing remains a major challenge. To address this, we analyzed T cell differentiation status, TCR Vβ repertoire, T cell exhaustion, and proliferative capacity in patient-derived samples collected within seven days prior to leukapheresis and correlated the results with product specifications. Consistent with published pediatric real-world data showing comparable efficacy and safety of OOS and in-specification tisagenlecleucel (18, 19), OOS status should not be equated with inferior clinical efficacy. Rather, its clinical relevance lies in potential treatment delays and restricted access if re-manufacturing is required.

Patients with prior HSCT exhibited higher levels of CD4^+^ T cells and CD4^+^ EMRA T cell subsets. While Verlardi et al. reported prior HSCT as a limitation on proliferative capacity (20), we did not observe a statistically significant association between prior HSCT and OOS outcomes; however, the cohort was not powered to exclude a clinically relevant effect.

In 2022, O’Reilly et al. developed a predictive model for successful apheresis that took into account the pre-collection CD3^+^ cell count (8). In our analysis, the CD3 T cell count at leukapheresis correlated with the final T cell amount in the CAR-T cell product. A notable aspect of our cohort is that leukapheresis was generally performed only after recovery of clinically acceptable peripheral blood T-cell counts. Consequently, our findings do not merely reflect insufficient T-cell numbers available for collection. Despite successful leukapheresis and adequate peripheral blood T-cell recovery, OOS manufacturing outcomes still occurred. This observation suggests that quantitative T-cell recovery and functional T-cell fitness may represent distinct biological concepts. Functional proliferation assays may therefore provide complementary information beyond routine lymphocyte or CD3 counts. Hinrichs et al. have proposed that a high frequency of naive T cells is critical for the success of adoptive immunotherapy, as these cells are capable of more robust expansion, an observation that has been supported by subsequent studies (21–24). Less differentiated T cells, particularly those that have not yet acquired full effector function, appear to be well suited as starting material (25). Fraietta et al. further reported that sustained remissions in CLL patients treated with tisagenlecleucel were associated with higher frequencies of CD27^+^CD45RO^-^CD8^+^ T cells, representing naïve or early memory phenotypes prior to CAR-T manufacturing (26). Current studies are investigating stem cell memory (SCM) T cells in connection with CAR-T cell production. They are considered to be a naive-like, antigen-experienced, and self-renewing populations (27). Like naive cells, SCM T cells are associated with strong proliferation capacity (28, 29). Similar findings were obtained by Qayed et al., who observed an association between successful tisagenlecleucel manufacturing and increased levels of naive T cells, SCM T cells, and less differentiated memory cells (9). Consistently, Cuffel et al. reported that a higher proportion of naïve/SCM CD8^+^ T cell subsets at the time of apheresis was associated with early and long-lasting responses to tisagenlecleucel in Non-Hodgkin lymphoma (30). While we were able to demonstrate that the proliferation capacity of T cells is associated with CAR-T cell product quality, no association between the proportion of naive T cells and conformance with the product specifications was found in our study For CD8^+^ EMRA T cells, however, moderate correlations with insufficient cell viability and total cell counts were observed, suggesting an adverse impact of terminally differentiated T cell populations on CAR-T cell manufacturing. The differentiation panel used in this study did not allow discrimination between naïve and stem cell memory T cells, which have previously been implicated in successful CAR-T cell manufacturing. Therefore, the contribution of TSCM cells could not be directly assessed.

The efficiency of T cell immunity in recognizing antigens is largely based on the diversity of the T cell receptor repertoire (31, 32). In our study, we demonstrated an association between specific TCR Vβ subsets and manufacturing deviations. Exploratory TCR Vβ analyses identified several associations which should be interpreted cautiously and require validation in larger cohorts. Given the exploratory nature of this study and the limited sample size, no formal correction for multiple testing was applied. Therefore, findings from the correlation analyses, particularly regarding individual TCR Vβ subsets, should be considered hypothesis-generating and require validation in independent cohorts. In addition, the decision-tree thresholds identified in the multivariate analyses are highly data-driven and should not be interpreted as clinically meaningful cut-offs, but rather as exploratory findings requiring confirmation in larger datasets.

Exhausted T cells are characterized by loss of effector functions, impaired proliferation, changes in cell metabolism, and reduced persistence (33–35). Dimitri et al. have proposed that the limited *in vivo* therapeutic efficacy of CAR-T cells in a subset of patients may in part be attributed to a pre-existing exhausted state of T cells (36). According to Wherry et al., the presence of multiple inhibitory receptors at the same time, rather than PD-1 alone, indicates a state of exhaustion (37, 38). In addition, previous studies have described a correlation between the number of inhibitory receptors and the severity of exhaustion (35, 37). Since none of the patients in our study displayed clear expression of all three markers considered, no severe T cell exhaustion could be detected according to the above definition.

Among all parameters analyzed, proliferation markers provided the strongest association with OOS events. Using a cut-off derived from healthy donors, poor *in vitro* proliferation was observed in approximately one third of patients, suggesting that impaired T-cell proliferative capacity is a frequent finding in this cohort and supporting further evaluation of this parameter prior to apheresis. In our study, poor T cell proliferative capacity was strongly associated with failure to meet the product specifications. This is in line with Baguet et al., who interpreted proliferative capacity as one of the key factors for successful production of CAR-T cells and as a predictive factor for *in vivo* expansion and activity (7). Furthermore, Cuffel et al. were able to link proliferative potential to clinical outcome. They evaluated the proliferation capacity of T cells from 30 patients with aggressive diffuse large B-cell lymphoma or transformed follicular lymphoma who underwent CAR-T cell therapy with tisagenlecleucel correlating progression-free survival with the *in vitro* proliferation potential of CD8^+^ T cells obtained from apheresis (30).

A further limitation is that proliferation was assessed using freshly isolated PBMCs, whereas tisagenleucel manufacturing is performed using cryopreserved leukapheresis products. Nevertheless, impaired proliferation was already detectable prior to collection, suggesting that reduced T-cell fitness may persist throughout subsequent manufacturing steps.

From a practical perspective, pre-apheresis assessment of T-cell proliferation may complement, but not replace, established parameters of leukapheresis readiness such as absolute lymphocyte and CD3+ T-cell counts, T-cell differentiation, the interval since the last lymphotoxic therapy and the patient’s clinical condition. As results are available before the collection date, a low DIL value could prompt clinicians, if the clinical situation allows, to postpone leukapheresis to permit further T-cell recovery and to repeat the assessment. In addition, a potential manufacturing delay could be taken into account when planning bridging therapy. Increasing the collection target is not a realistic option, as the target cell dose for tisagenlecleucel is already high, and repeated or back-up collections would not address impaired functional T-cell fitness. As no validated threshold can be derived from four OOS events, DIL should currently be regarded as one piece of an integrated assessment, and its clinical utility warrants prospective evaluation in larger cohorts.

In summary, identifying factors associated with manufacturing deviations CAR-T cell product quality is crucial, as these could assist clinicians in making complex management decisions for patients with refractory hematologic malignancies.

In this prospective multicenter pediatric study, impaired ex vivo T-cell proliferation before leukapheresis was associated with subsequent OOS tisagenlecleucel manufacturing outcomes. These findings support further evaluation of simple functional T-cell assays as complementary measures of leukapheresis readiness.

## Data Availability

The raw data supporting the conclusions of this article will be made available by the authors, without undue reservation.

## Supplementary material

Supplementary Figure 1 Univariate correlation of TCR Vβ repertoire, T cell differentiation, and exhaustion with CAR-T cell specifications. Additionally, the parameters age at apheresis, gender, and transplantation status were considered. Negative correlations are shown in red, positive correlations in blue. **(A)** TCR Vβ repertoire: Heatmap visualization of the correlation between individual Vβ subsets and the number of Vβ subsets outside the reference range (T4out, T8out) with product specifications (n = 22 patients) (left). Representative flow cytometry plots of all 24 Vβ subsets included in the panel covered by 8 separate measurements with distinct antibody combinations (middle). Schematic representation of TCR Vβ frequencies and their respective reference range (right).

Supplementary Figure 2 Absolute T-cell counts in patients with and without prior HSCT. Absolute counts of CD3^+^, CD3^+^CD4^+^ and CD3^+^CD8^+^ T cells (cells/µL) in peripheral blood of patients with (hatched bars, circles; n = 17) and without (open bars, diamonds; n = 10) prior allogeneic HSCT. Bars represent medians, whiskers indicate the range, and symbols represent individual patients. Differences between groups were not statistically significant (two-tailed Mann–Whitney U test).
